# Single-cell landscape of immune remodeling in alopecia areata suggests MIF + fibroblasts and their potential ligand-receptor crosstalk with dendritic cells

**DOI:** 10.3389/fmed.2026.1849368

**Published:** 2026-05-22

**Authors:** Xuemei Lan, Haiyan Li, Yunting Xiao

**Affiliations:** 1 Department of Dermatology, Zigong Fourth People’s Hospital, Zigong, Sichuan, China; 2 Clinical Laboratory, Qingdao Traditional Chinese Medicine Hospital, Qingdao Hiser Hospital Affiliated of Qingdao University, Qingdao, China

**Keywords:** alopecia areata, fibroblasts, hair follicle epithelium, hair follicle stem cells, single-cell RNA sequencing

## Abstract

**Background:**

Alopecia areata (AA) is an autoimmune hair loss disorder. Although autoreactive CD8^+^ T cells are central effectors, the roles of stromal and epithelial compartments in sustaining inflammation remain unclear.

**Methods:**

This study analyzed publicly available scRNA-seq data from the GEO database (GSE212450, GSE233906), comprising 15 scalp samples from normal skin (NS), non-lesional (NL), and AA conditions. Following integration, normalization, and clustering, cells were annotated using established marker genes. Subclustering focused on immune, epithelial, and fibroblast lineages. Differential expression and enrichment analyses identified disease-associated genes and pathways. CellChat was used to compare ligand–receptor interactions and signaling networks between NS and AA microenvironments.

**Results:**

scRNA-seq analysis revealed a profoundly remodeled immune niche in AA lesions, extending beyond immune cells to encompass epithelial cells and fibroblasts. Key features included lymphoid-myeloid expansion, clonal cytotoxic CD8^+^ T1 cell accumulation, mast cell loss, and elevated DC3-derived IL-15. Epithelial cells exhibited upregulated MHC-II and immunomodulatory activity despite stable proportions. A novel pro-inflammatory fibroblast subset (FB3), enriched in AA, expressed high levels of MIF, displayed inflammatory and stress-related signatures, and suppressed extracellular matrix organization. Ligand-receptor analysis identified an intensified MIF-centered inflammatory circuit, primarily via MIF-(CD74 + CXCR4/CD44) axes, forming a robust signaling network connecting FB3 and multiple dendritic cell subsets.

**Conclusion:**

This study reveals that fibroblasts in AA lesions, particularly the newly identified FB3 subset, are not merely passive structural cells but rather active participants in immune-inflammatory regulation through the elevated secretion of MIF. The enhanced MIF-(CD74 + CXCR4/CD44) signaling axis constitutes a core mechanism connecting FB3 cells with multiple dendritic cell subsets. This finding suggests that targeting FB3 differentiation or inhibiting MIF-related signaling pathways may represent a promising therapeutic avenue for AA.

## Introduction

Alopecia areata (AA) is a complex immune-mediated disorder characterized by the loss of immune privilege in the hair follicle, leading to non-scarring hair loss. The pathogenesis is primarily driven by a collapse of the hair follicle’s immune privilege, enabling autoreactive CD8^+^ T cells to recognize follicular autoantigens (1). These cytotoxic T cells, often supported by T helper cells and natural killer cells, infiltrate the hair follicle bulb and are believed to be the primary executors of hair follicle dystrophy and arrested growth. A key mechanistic pathway involves interferon-gamma (IFN-*γ*) signaling, which upregulates major histocompatibility complex (MHC) class I and II expression on hair follicle epithelium and activates the JAK–STAT pathway, thereby amplifying the inflammatory cascade (2).

A challenging clinical aspect of AA is its frequent recurrence following successful treatment or even spontaneous remission. The mechanisms underlying this recurrence remain poorly understood but may involve the persistence of a pathological cellular “memory” (3). Hypotheses include the survival of autoreactive T-cell clones, the formation of resident memory T cells within the scalp skin that are reactivated upon unknown triggers, and the failure to fully restore a functional immune privilege in “recovered” hair follicles (4).

Crucially, the transition from a normal scalp to an AA-affected state involves a dramatic reorganization of the local inflammatory microenvironment. However, our current understanding of this microenvironment is largely centered on the adaptive immune response (5). The role of non-immune stromal cells, such as fibroblasts, and the dynamic changes in other cell populations during disease initiation and progression are less defined. It is plausible that these resident cells are not merely passive targets but active participants in creating and sustaining the inflammatory niche that drives AA pathology. Single-cell RNA sequencing (scRNA-seq) has emerged as a powerful tool for deconvoluting the cellular heterogeneity and complex cell–cell interactions within inflammatory microenvironments of various diseases (6). Previous scRNA-seq studies in inflammatory skin diseases have successfully mapped cellular heterogeneity and implicated even non-inflammatory skin resident cells in disease pathogenesis. For instance, in psoriasis, scRNA-seq studies have revealed novel fibroblast and dendritic cell subsets that promote T-cell activation and chronic inflammation (7). Similarly, in rheumatoid arthritis, this technology has identified pathogenic synovial fibroblast subpopulations that mediate bone erosion and resist treatment (8). Research by Chen’s team demonstrated that fibroblasts play an essential role in vitiligo pathogenesis. Their work identified a pivotal mechanistic interplay between fibroblasts and CD8^+^T cells underlying this autoimmune condition (9). Therefore, this study analyzed publicly available scRNA-seq datasets to profile the inflammatory microenvironment of AA lesions vs. healthy scalp skin. The objective was to identify transcriptionally reprogrammed non-immune resident cells—including fibroblasts and epithelial cells—that contribute to sustained inflammation, moving beyond immune characterization to inform new therapeutic avenues.

## Methods

### Single-cell RNA sequencing data acquisition and analysis

Publicly available scRNA-seq data were downloaded from the Gene Expression Omnibus (GEO) database (accession numbers: GSE212450 and GSE233906). Publicly available raw sequencing data were obtained for 15 samples: GSM6532919, GSM6532920, GSM6532921, GSM6532922, GSM6532923, GSM6532924, GSM6532925 (GSE212450) and GSM7438941-GSM7438948 (GSE233906). Samples were categorized into three clinical conditions: NS (normal scalp), NL (non-lesional scalp), and AA. Among these, GSM6532919, GSM6532920, GSM6532921, GSM6532922, GSM7438941, GSM7438942, GSM7438943, GSM7438944, GSM7438945 and GSM7438948 are from AA; GSM7438946 and GSM7438947 are from NL; and GSM6532923, GSM6532924, and GSM6532925 are from NS. This classification enabled comparative analyses across different disease states, with the condition variable incorporated as a key metadata attribute for all downstream investigations. Data analysis was performed using Seurat (v4.4.0) in R (v4.3.2). Briefly, cells were filtered based on the following stringent criteria: mitochondrial gene percentage < 20%, hemoglobin gene percentage < 1%, and the number of detected genes between 200 and 4,000. This rigorous filtering process resulted in a high-quality dataset comprising 76,727 cells and 25,356 genes for subsequent analysis. Following quality control, the SCTransform method was employed for normalization and variance stabilization, with 4,000 highly variable features selected for downstream analysis. To address potential batch effects across different samples, we applied Harmony integration with the following parameters: lambda = 2, maximum iterations = 20, using the “orig.ident” variable as the batch covariate. Principal component analysis (PCA) was performed on the integrated data, with the first 50 principal components selected for subsequent analyses based on elbow plot evaluation. Uniform Manifold Approximation and Projection (UMAP) was utilized for two-dimensional visualization using the Harmony-corrected dimensions. Cell clustering was performed using the FindNeighbors and FindClusters functions with a resolution parameter of 0.5, resulting in distinct cell populations. Cell clusters were annotated based on the expression of well-established cell-type-specific marker genes. This integrated analytical pipeline enabled comprehensive characterization of the cellular landscape and transcriptional profiles across different skin conditions at single-cell resolution.

### Subclustering of major cell lineages

To delineate cellular heterogeneity within key lineages implicated in AA pathogenesis, fibroblasts, keratinocytes, and immune cells were isolated from the integrated single-cell atlas for subsequent deep, lineage-specific re-analysis. Each major cell population was subsetted and subjected to an independent analytical pipeline consistent with the primary analysis. This included re-normalization using SCTransform with regression of mitochondrial gene expression, data integration via Harmony to correct for batch effects, and subsequent dimensionality reduction, clustering, and UMAP visualization. The resolution parameter for clustering was empirically optimized for each lineage to best capture biologically distinct subpopulations. Annotation of cell subtypes was performed based on the expression of well-established marker genes from the literature, allowing for the identification of functionally specialized states within each major lineage.

### Differential expression and cell proportion analysis

Differential expression analysis was conducted to identify genes with significant alterations in expression across distinct cell populations and conditions. For comparisons between pre-defined cell clusters, the Wilcoxon rank-sum test was implemented using the FindMarkersfunction in Seurat, with a threshold of absolute log₂-fold change (|log₂FC|) > 0.25 and minimum expression percentage > 10% in either population. To ensure statistical rigor in group-wise comparisons, a more stringent cutoff was applied, requiring |log₂FC| > 0.5 and an adjusted *p*-value (p.adj) < 0.05 for significance. This two-tiered approach allowed for robust identification of both cell-type-specific markers and condition-associated differentially expressed genes. Given the small sample size of the NL group, all statistical comparisons of cell proportions—including those of immune cells, fibroblasts, and epithelial-derived cells—were performed only between the NS and AA groups using the same Wilcoxon rank-sum test.

### Functional enrichment analysis

To interrogate the biological functions of the differentially expressed genes (DEGs), Gene Ontology (GO) and Kyoto Encyclopedia of Genes and Genomes (KEGG) pathway enrichment analyses were conducted using the compareCluster function from the clusterProfiler R package. This allowed for simultaneous comparison of functional profiles across multiple clusters. For Gene Set Enrichment Analysis (GSEA), a ranked gene list was generated based on the log2-fold change derived from the differential expression test. Pre-ranked GSEA was then performed using the clusterProfiler and GseaVis packages against the GO Biological Processes (BP) gene set collection from the MSigDB database to identify signaling pathways enriched in a coordinated manner.

### Cell–cell communication analysis

Intercellular signaling networks were systematically analyzed to identify communication alterations associated with disease states using the CellChat package (v1.6.1). This computational framework employs a probabilistic model to infer communication probabilities between cell populations by integrating scRNA-seq expression data with a curated ligand-receptor interaction database. The analysis was performed on the integrated dataset containing keratinocytes, fibroblasts, and immune cells from both NS and AA conditions. The dataset was partitioned by condition, and CellChat objects were created separately for NS and AA samples using normalized expression data from the RNA assay and cell type annotations from Seurat metadata. The human ligand-receptor interaction database (CellChatDB.human) was used as the reference set. The analytical pipeline involved three principal steps: first, identification of over-expressed ligands and receptors within each cell type; second, computation of communication probabilities for each ligand-receptor pair, accounting for relative expression levels and pathway co-expression patterns; and third, aggregation of individual ligand-receptor pairs to infer pathway-level communication networks. To ensure robustness, interactions supported by fewer than 10 cells in any cell group were excluded. Social network analysis was further applied to calculate network centrality measures, thereby identifying key signaling roles in the communication networks. For comparative analysis between conditions, the NS and AA CellChat objects were merged to enable systematic evaluation through three approaches: (1) comparison of interaction numbers and weights, (2) differential interaction analysis to identify significantly altered communications, and (3) pathway-level enrichment analysis to detect condition-specific signaling pathways. Multiple visualization methods including circle plots, heatmaps, bubble charts, and hierarchical plots were utilized to represent the complex communication networks. Specific pathways of interest, such as MIF signaling, were examined in detail to elucidate their potential roles in disease pathogenesis. Validation of inferred communications was performed through multiple approaches: examination of gene expression patterns for key ligands and receptors across conditions, subset analysis focusing on specific cell-type interactions (e.g., fibroblast-immune cell crosstalk), and statistical testing to identify significantly different interactions. All visualizations were generated using CellChat’s built-in functions with consistent color schemes and scaling to ensure comparability. This comprehensive analysis provided insights into the rewiring of cell–cell communication networks in AA, revealing both conserved and disease-specific signaling patterns among major skin cell types.

### Immunofluorescence quantification

To quantify the enrichment of FB3-like (Vimentin^+^MIF^+^) fibroblasts, dual-color immunofluorescence staining for Vimentin and MIF was performed on scalp tissue sections from normal and AA lesional samples. Images were acquired under identical acquisition settings. For each sample, at least three non-overlapping fields were randomly selected. Within each field, the total number of Vimentin^+^ cells (spindle-shaped, cytoplasm-rich cells, excluding round immune-like cells) was counted, and the number of Vimentin^+^MIF^+^ double-positive cells was counted using ImageJ/Fiji. The percentage of double-positive cells among total Vimentin^+^ cells was calculated per field and averaged per sample. Data are presented as bar charts with individual sample points, and statistical comparison between normal and AA groups was performed using the Mann–Whitney U test. A *p* value < 0.05 was considered significant.

## Results

### Integrated analysis unveils a pro-inflammatory niche in alopecia areata: overall lympho-myeloid expansion highlighting CD8 ^+^T cells and diminished mast cells

**Figure 1:**
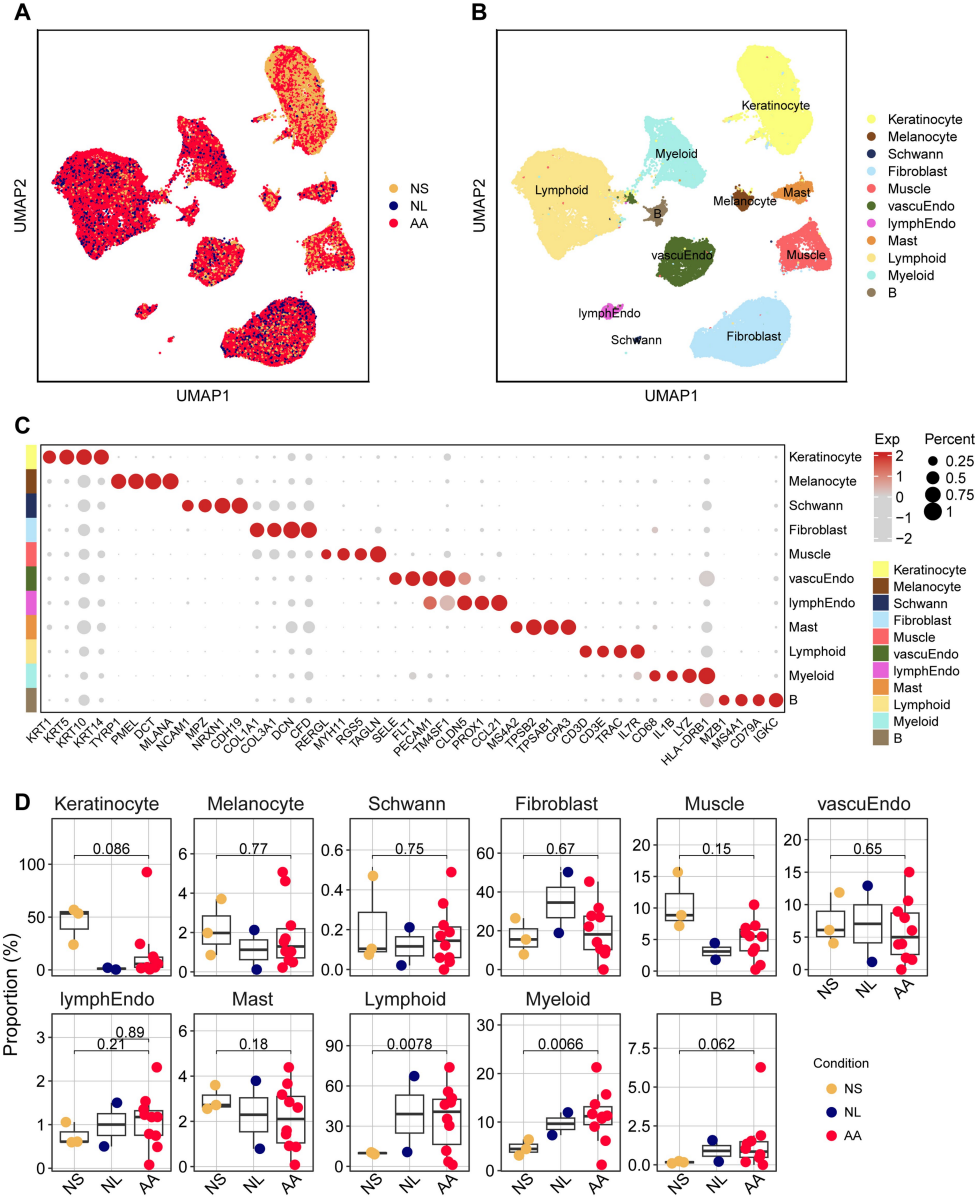
Characterization of cell clusters from NS, NL, and AA. **(A)** Integration of scRNA-seq datasets from NS, NL, and AA (yellow: NS; blue: NL; red: AA). **(B)** UMAP visualization of all cell clusters identified across NS, NL, and AA samples, with distinct colors representing different cell clusters. **(C)** Dot plot showing the expression of distinctive markers for each cell population. **(D)** Bar charts showing the fold changes in cell proportions for all cell clusters across NS, NL, and AA.

To map the cellular landscape of AA, we integrated public scRNA-seq data from NS, NL, and AA. A final set of 76,727 high-quality cells from the NS, NL and AA groups were retained after filtering out doublets and low-quality cells (Supplementary Figures S1A,B). Unsupervised clustering identified 11 major cell lineages, which were annotated based on well-characterized lineage-specific markers: keratinocytes (Keratinocyte; KRT1, KRT5, KRT10, KRT14); melanocytes (Melanocyte; TYRP1, PMEL, DCT, MLANA); schwann cells (Schwann; NCAM1, MPZ, NRXN1, CDH19); fibroblasts(Fibroblast; COL1A1, COL3A1, DCN, CFD); muscle cells (Muscle; RERGL, MYH11, RGS5, TAGLN); vascular endothelial cells (vascuEndo; SELE, FLT1, PECAM1, TM4SF1); lymphatic endothelial cells (lymphEndo; PECAM1, CLDN5, PROX1, CCL21); mast cells(Mast; MS4A2, TPSB2, TPSAB1, CPA3); lymphoid cells (Lymphoid; CD3D, CD3E, TRAC, IL7R); myeloid cells (Myeloid)(CD68, IL1B, LYZ, HLA-DRB1); and B cells (B; MZB1, MS4A1, CD79A, IGKC; Figures 1A–C). UMAP plot revealed cell clusters and displayed the expression levels of their canonical marker genes (Supplementary Figure S1C). Comparative analysis revealed a significant lymphoid and myeloid cell infiltration in AA lesions, with a 2.5-fold increase (*p* < 0.05) compared to NS (Figure 1D). No statistically significant differences were observed among the two groups—NS, and AA—in terms of the proportions of keratinocytes and fibroblasts (Figure 1D).

**Figure 2:**
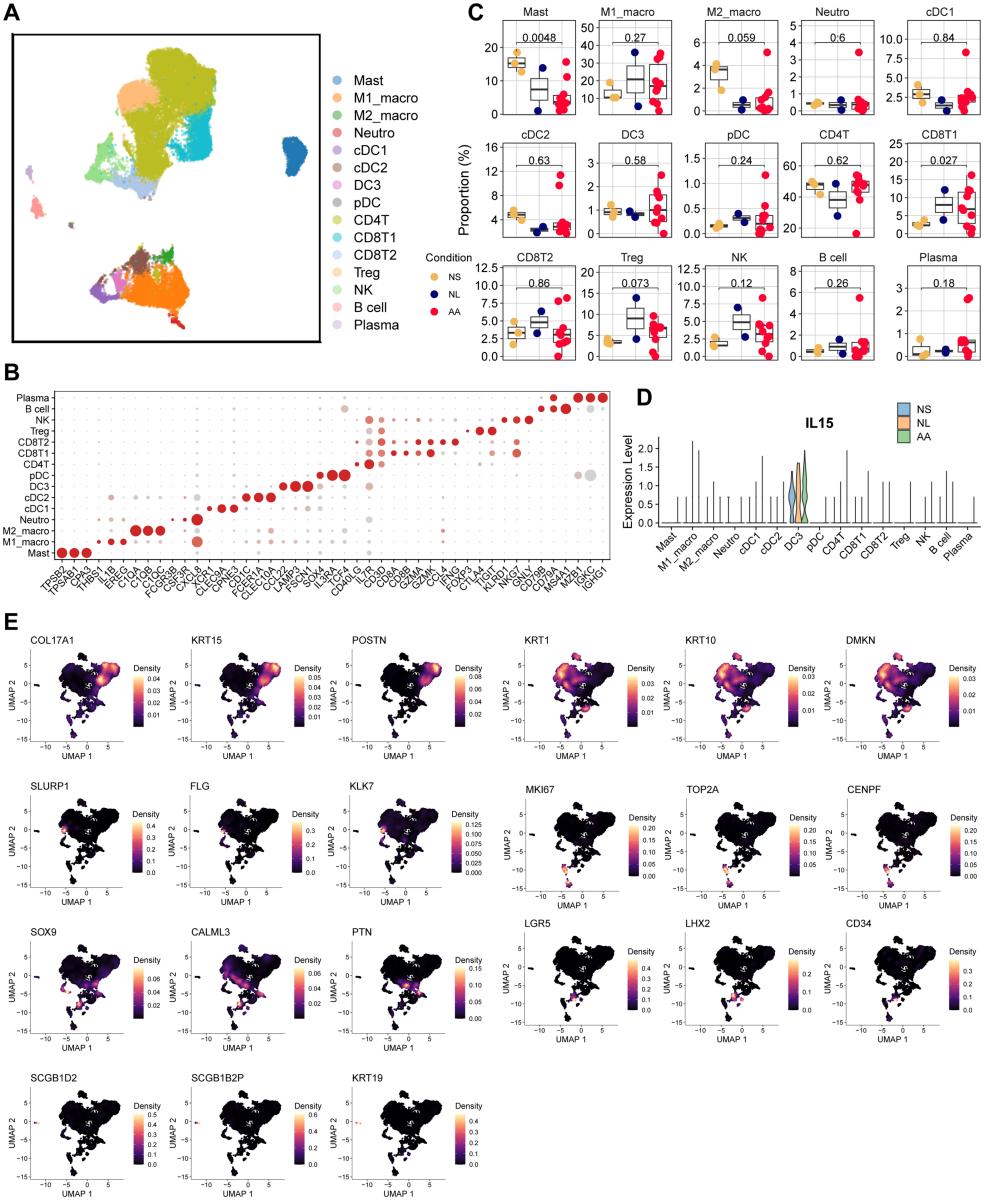
Comprehensive analysis of immune and epithelial cell subpopulations. **(A)** Identification of 15 immune cell subsets across NS, NL, and AA specimens. **(B)** Bubble plot showing the top three marker genes for each immune cell subpopulation. **(C)** Comparison of immune cell proportions among NS, NL, and AA. **(D)** Comparative analysis of IL-15 secretion patterns among immune cell subsets in NS, NL, and AA. **(E)** UMAP visualization of the top three marker genes for seven epithelial-derived cell clusters.

To dissect the immune compartment in detail, we subset and re-clustered all immune cells (mast cell, myeloid cells, and lymphoid cells), identifying 15 distinct subpopulations (Figure 2A). The top three marker genes for each cluster were identified using the FindAllMarkers function and visualized in a bubble plot. Based on the expression patterns of these marker genes and established cell-type signatures from the literature, all clusters were classified into the following 15 immune cell types: mast cells(Mast; marked by TPSB2, TPSAB1, CPA3), M1 macrophages (M1_macro; THBS1, IL1B, EREG, CXCL8), M2 macrophages (M2_ macro; C1QA, C1QB, C1QC), neutrophils (Neutro; FCGR3B, CSF3R, CXCL8), type 1 conventional dendritic cells (cDC1; XCR1, CLEC9A, CPNE3), type 2 conventional dendritic cells (cDC2; CD1C, FCER1A, CLEC10A), type 3 dendritic cell (DC3; CCL22, LAMP3, FSCN1), plasmacytoid dendritic cells (pDCs; SOX4, IL3RA, TCF4), CD^+^4 T cells (CD4T; CD40LG, IL7R, CD3D), CD^+^8 T1 cells (CD8T1; CD8A, CD8B, GZMA, GZMK), CD^+^8 T2 cells (CD8T2; GZMA, CCL4, IFNG), Tregs (Treg; FOXP3, CTLA4, TIGIT), NK cells (NK; KLRD1, NKG7, GNLY), B cells (B; CD79B, CD79A, MS4A1), and plasma cells (Plasma; MZB1, IGKC, IGHG1; Figure 2B). A significant reduction in mast cells alongside a concomitant expansion of CD8T1 cells was observed in AA lesions (Figure 2C). This reduction was not evident in global clustering, likely because the large influx of other immune cells in AA dilutes mast cell proportions. Notably, among all cell types—including epithelial-derived cells, immune cells, and stromal cells—IL-15 was found to be mainly secreted by DC3 cells (LAMP3^+^, FSCN1^+^; Supplementary Figure S2). Specifically, the DC3 subpopulation exhibited heightened IL15 expression compared to other immune cells across all conditions (NS, NL, AA; Figure 2D). Moreover, this elevated IL-15 expression was further accentuated in AA compared to NS (Figure 2D).

### Epithelial transcriptional signatures in alopecia areata: immune activation and stem cell dysregulation

**Figure 3:**
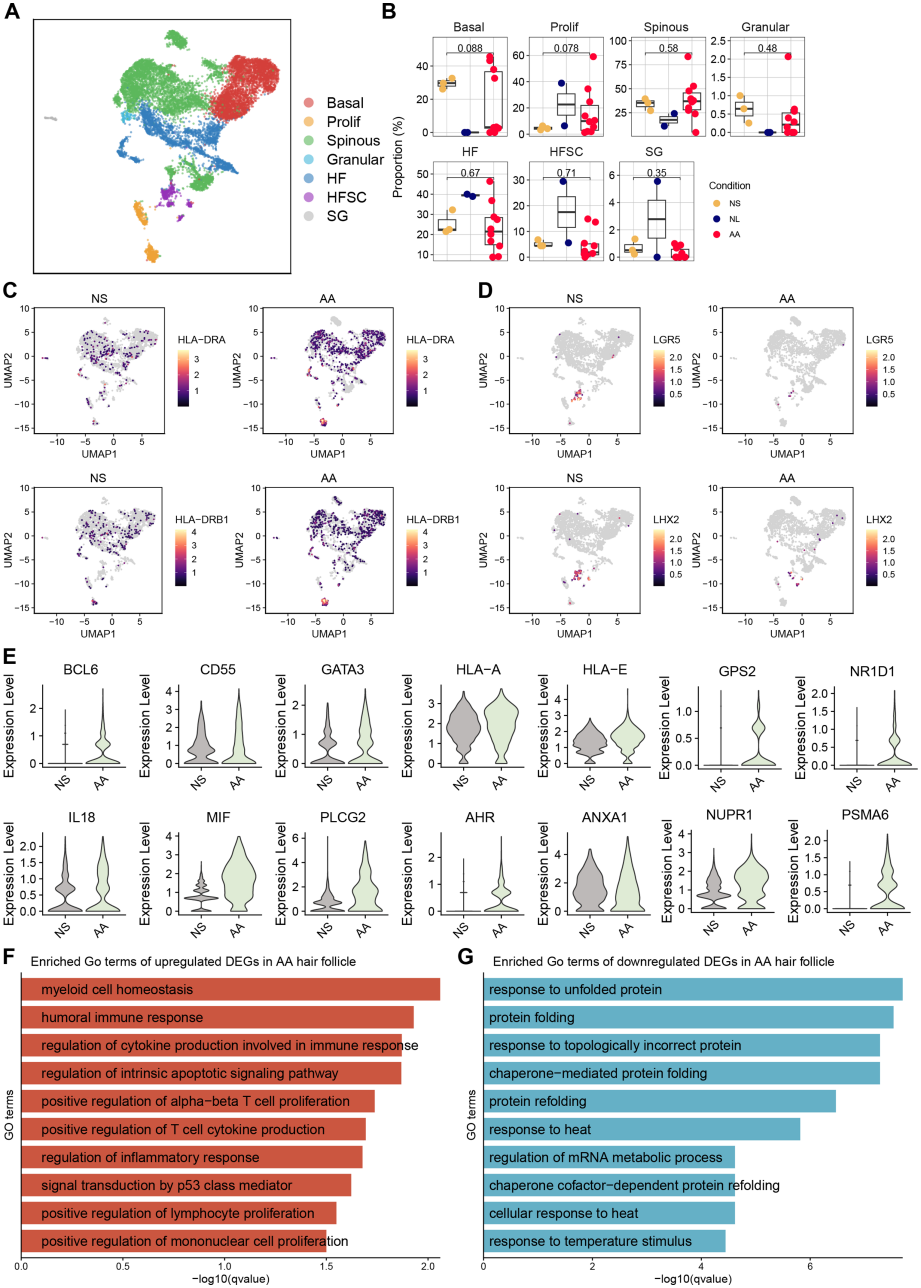
Epithelial cells acquire immune-like features in AA. **(A)** Seven distinct epithelial-derived cell subpopulations identified across NS, NL, and AA specimens. **(B)** Comparison of epithelial-derived cell proportions across NS, NL, and AA. **(C)** UMAP visualization showing differential expression of HLA-DRA and HLA-DRB1 between AA and NS. **(D)** UMAP visualization showing differential expression of LGR5 and LHX2 between AA and NS. **(E)** Violin plots depicting differential enrichment of inflammatory pathway genes between AA and NS. **(F)** GSEA analysis showing the top 10 significantly upregulated GO terms based on differentially expressed genes in hair follicles between AA and NS. **(G)** GSEA analysis showing the top 10 significantly downregulated GO terms based on differentially expressed genes in hair follicles between AA and NS.

Subsequent analysis focused on the epithelial compartment, identifying seven distinct keratinocyte subpopulations: basal keratinocytes (Basal; marked by KRT17A1, KRT15 and POSTN), proliferating keratinocytes (Prolif; MKI67, TOP2A, CENPF), spinous keratinocytes(Spinous; KRT1, KRT10, DMKN), granular keratinocytes (Granular; SLURP1, FLG, KLK7), hair follicle keratinocytes (HF; SOX9, CALML3, PTN), hair follicle stem cells (HFSC; LGR5, LHX2, CD34), and sebaceous gland keratinocytes (SG; SCGB1D2, SCGB1B2P, KRT19; Figures 2E, 3A). Despite showing a largely preserved cellular composition compared to NS (Figure 3B), the epithelial compartment in AA exhibited striking transcriptional changes. These included a marked upregulation of MHC class II genes (HLA-DRA, HLA-DRB1) in basal, spinous, granular, HF and HFSC, alongside a marked downregulation of LGR5 and LHX2 in HFSCs (Figures 3C,D). In AA, epithelial-derived cells demonstrated a significant increase in the expression of inflammatory-associated factors—such as HLA-A, HLA-E, MIF, PLCG2, and NUPR1—compared to NS (Figure 3E). Functional enrichment analysis confirmed a broad shift in the functional state of HF, characterized by the activation of immune-related processes alongside the suppression of protein homeostasis pathways. Notably, representative upregulated pathways included myeloid cell homeostasis, humoral immune response, and regulation of cytokine production, while downregulated ones featured processes such as response to unfolded protein, protein folding, and response to topologically incorrect protein (Figures 3F,G).

### Identification of a pathogenic, MIF-expressing inflammatory fibroblast subset

**Figure 4:**
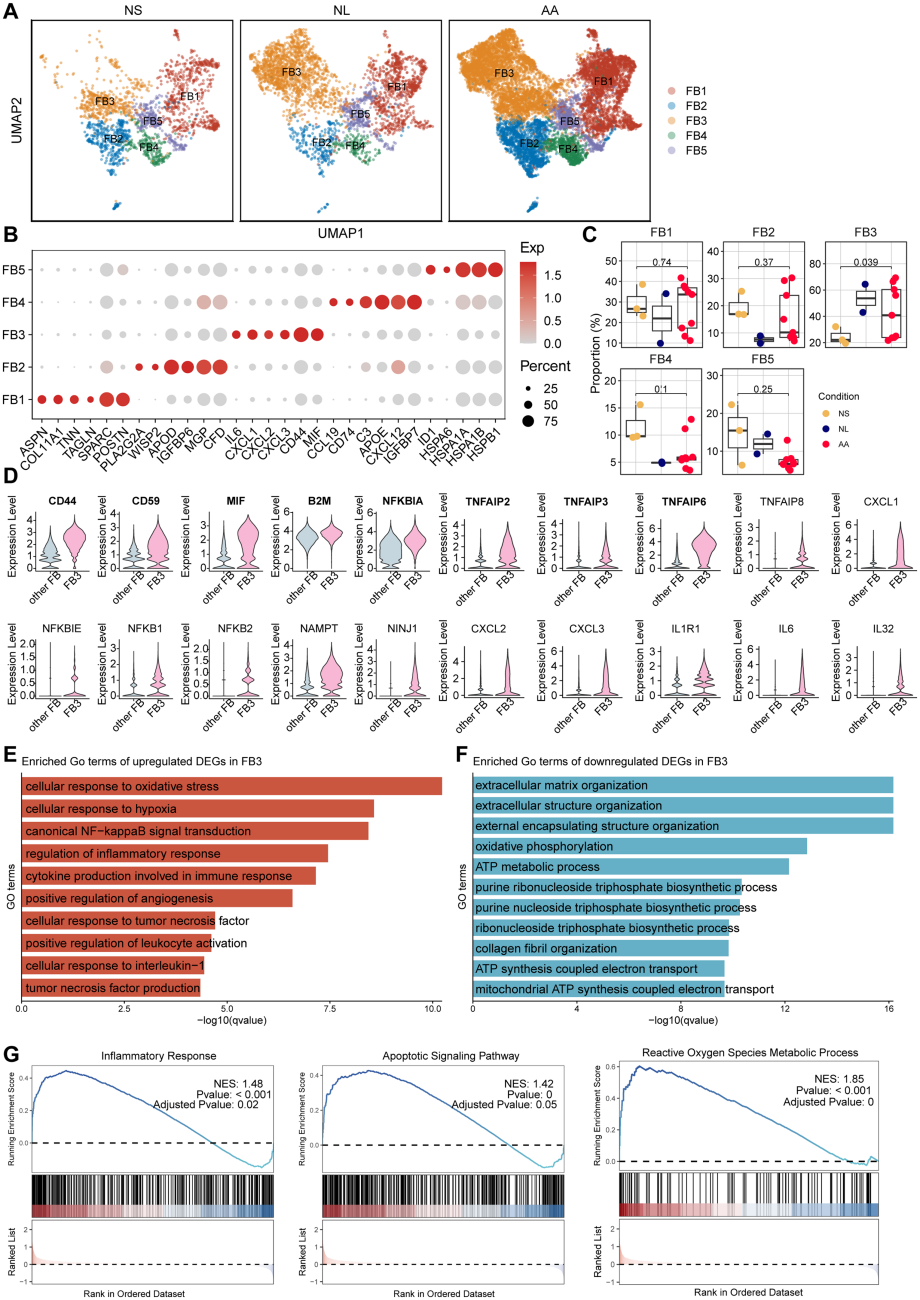
Novel fibroblast subpopulation in AA. **(A)** UMAP visualization of fibroblast subtype distribution across NS, NL, and AA. **(B)** Bubble plot showing marker genes of different fibroblast subpopulations. **(C)** Comparison of fibroblast subpopulation proportions among NS, NL, and AA. **(D)** Top 20 differentially expressed genes in FB3 compared with other fibroblast subpopulations. Genes mentioned in the text (CD44, CD59, MIF, B2M, NFKBIA, TNFAIP2, TNFAIP3, TNFAIP6) are shown in bold in the figure. **(E)** GSEA analysis showing significantly upregulated GO terms between FB3 and other fibroblasts in AA. **(F)** GSEA analysis showing significantly downregulated GO terms between FB3 and other fibroblasts in AA. **(G)** Functional enrichment of upregulated pathways in AA-derived FB3 fibroblasts, depicting the inflammatory response, apoptotic signaling pathway, and reactive oxygen species (ROS) metabolic process.

**Figure 5:**
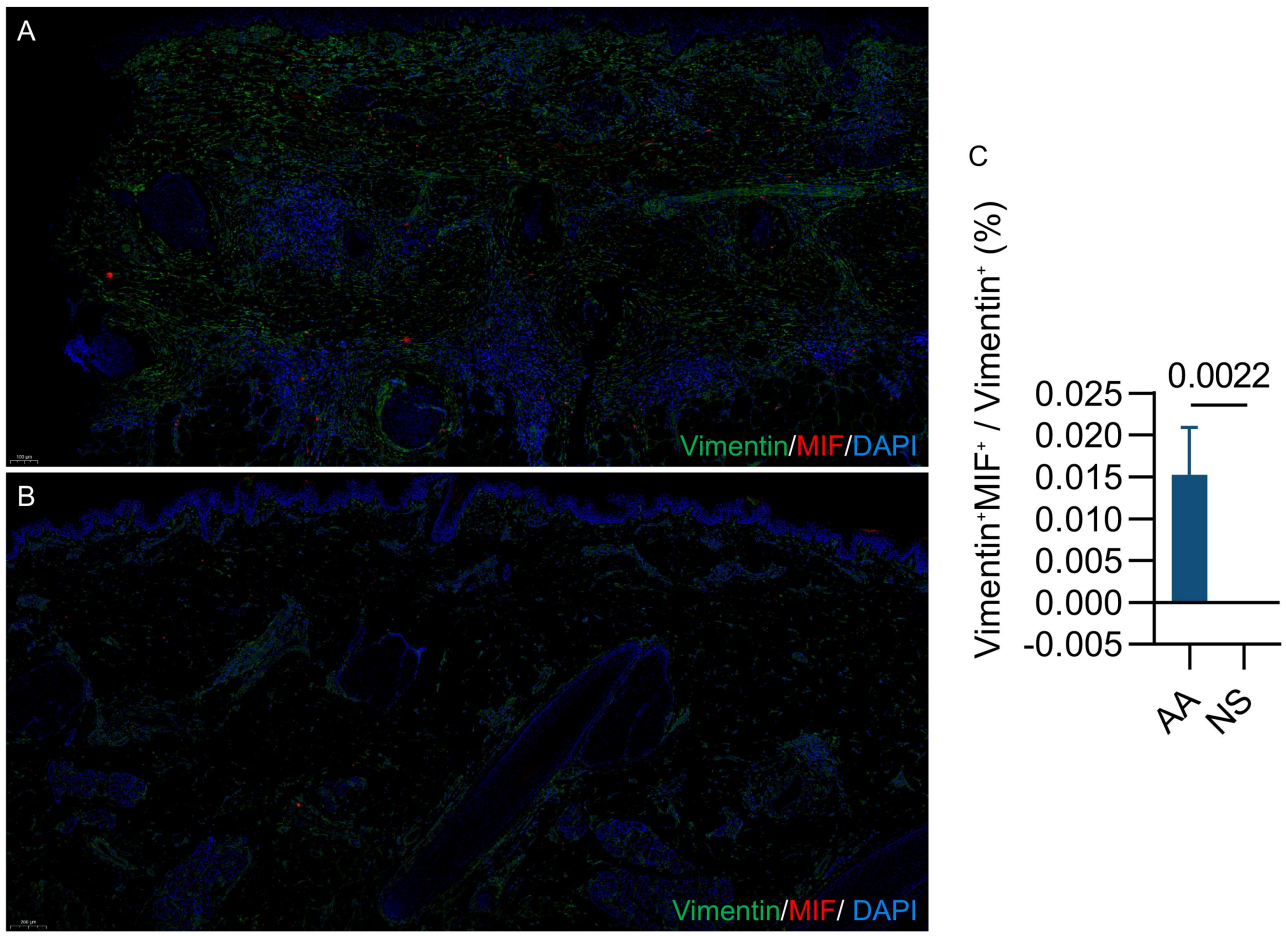
Differential expression of MIF-positive fibroblasts in AA vs. NS. **(A)** MIF expression in fibroblasts from AA tissue. Immunofluorescence staining for fibroblasts (vimentin, green), MIF (red), and nuclei (DAPI, blue). **(B)** MIF expression in fibroblasts from NS tissue. **(C)** Enrichment of Vimentin^+^MIF^+^ cells in AA lesions compared to NS. Statistical analysis was performed using the Mann–Whitney *U* test, and a *p*-value < 0.05 was considered significant.

Analysis of the stromal compartment revealed the consistent identification of five distinct fibroblast subpopulations (FB1-FB5) across all sample groups: NS, NL and AA (Figures 4A,B). Relative to NS, the proportion of FB3 (defined by high CD44 and MIF expression) was significantly increased in AA lesions (*p* < 0.05; Figures 4B,C). To further confirm the stromal cell characteristics of FB3, the distribution of PI16 and SCARA5 across the five fibroblast subtypes was re-examined, revealing that PI16 is broadly expressed across all fibroblast subsets, while SCARA5 is predominantly enriched in FB3 and FB2 (Supplementary Figures S3A,B). In contrast to other fibroblasts, this subset exhibited a potent inflammatory phenotype, distinguished by a marked upregulation of key mediators such as CD44, CD59, MIF, B2M, KFKBIA, TNFAIP2, TNFAIP3 and TNFAIP6 (Figure 4D). To elucidate the functional contribution of FB3 to AA, GSEA was conducted comparing FB3 with other fibroblasts. GSEA analysis revealed a distinct phenotypic shift in AA-derived FB3, characterized by a concurrent dysregulation of immune and structural pathways. This shift involved the upregulation of immune-inflammatory and stress-response pathways (e.g., inflammatory response, NF-κB signaling, apoptotic signaling, and reactive oxygen species metabolic process; Figures 4E,G), coupled with the downregulation of pathways responsible for extracellular matrix organization (Figure 4F). Immunofluorescence co-staining revealed that fibroblasts in AA exhibited increased expression of MIF compared to those in NS (Figures 5A,B). The proportion of Vimentin^+^ MIF double-positive cells was significantly greater in AA than in NS (*p* = 0.0022; Figure 5C).

### MIF + fibroblasts act on dendritic cells via the MIF/CD74 axis

**Figure 6:**
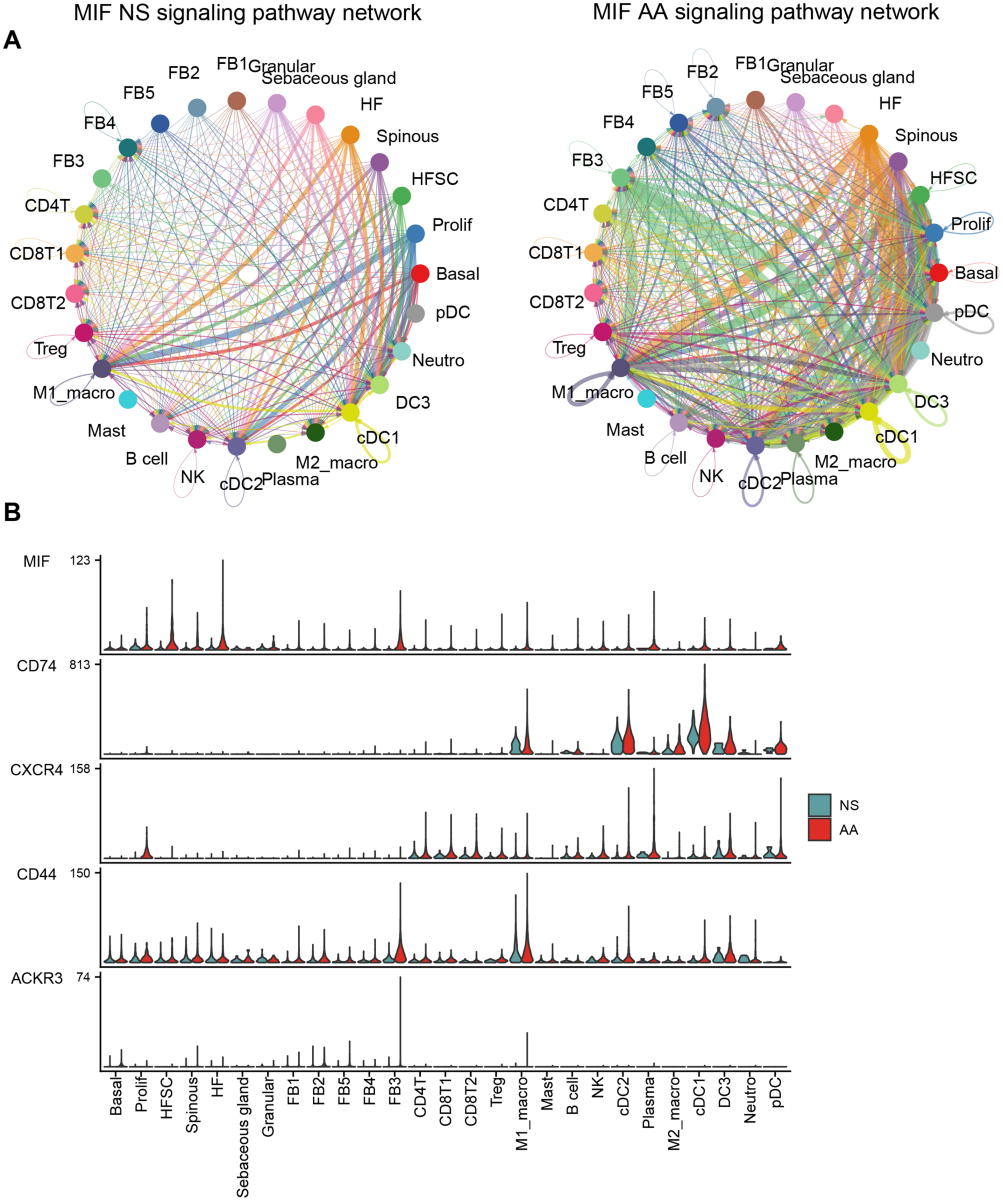
Interactions among immune cells, fibroblasts, and epithelial-derived cells in AA. **(A)** Comparative strength of MIF signaling pathway activity in fibroblast, immune cell, and epithelial subpopulations between AA and NS. **(B)** Differential expression of MIF pathway ligands and receptors in immune cell, fibroblast, and epithelial subpopulations between AA and NS.

Cell–cell interaction analysis revealed that, compared to normal scalp, FB3 (MIF + fibroblasts) in AA exhibited significantly enhanced interactions with multiple dendritic cell subsets—including M1-like macrophages, cDC1, cDC2, and DC3—via the MIF signaling pathway (Figure 6A). MIF exerts its biological functions through binding to multiple receptors, including the primary receptor CD74, the alternative receptors CXCR4 and CD44, and the atypical chemokine receptor ACKR3 (also known as CXCR7). To elucidate the cellular targets of MIF in AA, the expression patterns of these receptors across immune, stromal and epithelial cell populations were analyzed.

**Figure 7:**
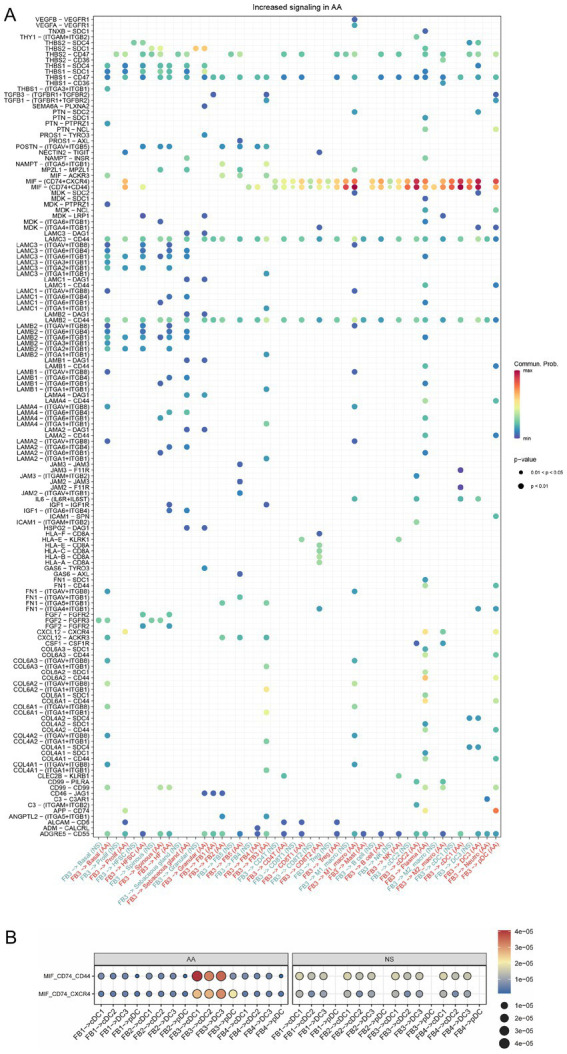
Differential ligand-receptor interactions between fibroblast subpopulations and target cells in AA vs. NS. **(A)** Bubble plot showing differential strength of ligand-receptor interactions from FB3 fibroblasts to target epithelial cells and immune cell subpopulations between AA and NS. **(B)** Bubble plot showing differential strength of ligand-receptor interactions between five fibroblast subtypes and dendritic cells (DCs).

**Figure 8:**
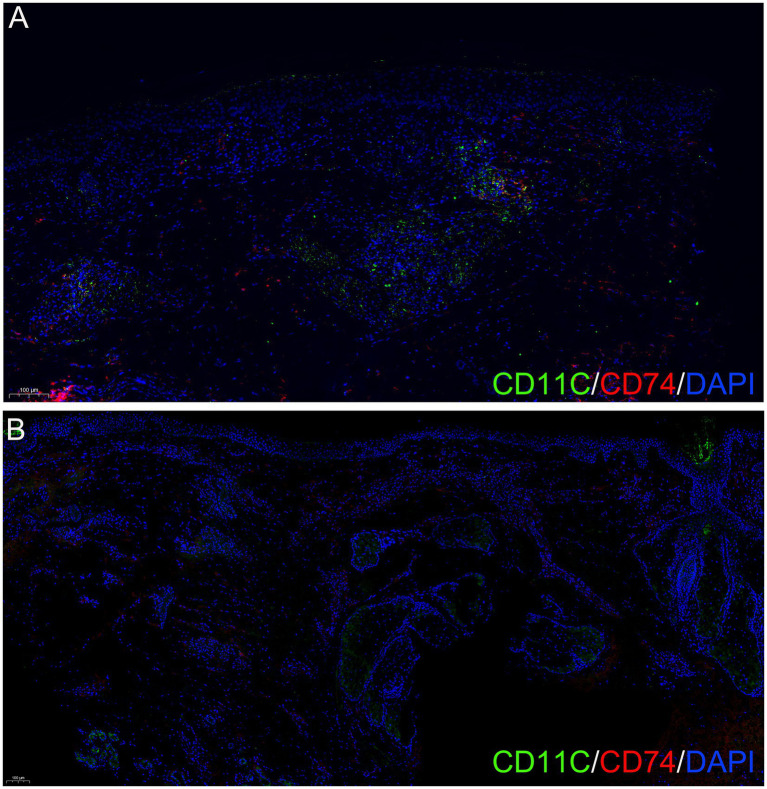
Elevated CD74 expression in dendritic cells from AA lesions compared to normal scalp. **(A)** CD74 expression in dendritic cells from AA lesions. **(B)** CD74 expression in dendritic cells from normal scalp. Immunofluorescence staining for dendritic cells (CD11c, green), CD74 (red), and nuclei (DAPI, blue).

The primary receptor CD74 was broadly distributed across dendritic cell subsets and was significantly upregulated in AA compared to normal scalp, particularly in M1-like macrophages, cDC1, cDC2, and DC3. In contrast, the alternative receptors CXCR4 and CD44 showed consistently low expression levels across all examined cell types in both conditions, with no significant differences between AA and normal scalp. Similarly, ACKR3 expression was barely detectable in both AA and normal scalp tissues, suggesting it does not play a major role in MIF signaling within the AA microenvironment (Figure 6B). Further ligand-receptor analysis suggested that FB3 primarily acts on various inflammatory cells through the MIF-CD74 + CXCR4 or MIF-CD74 + CD44 axes, with dendritic cells emerging as major targets (Figure 7A). Given that MIF is also expressed in other fibroblast subtypes, the interactions of five fibroblast subtypes with dendritic cells via the MIF signaling pathway (MIF-CD74 + CXCR4 or MIF-CD74 + CD44 axes) were examined to further elucidate the contribution of FB3 cells. The interactions of FB3 with cDC1, cDC2, DC3, and M1_macro appeared to be enhanced relative to normal scalp, and these interactions seemed to contribute to a greater extent to the MIF signaling pathway (Figure 7B). Immunofluorescence staining confirmed the presence of CD11c (dendritic cell surface marker) and CD74 double-positive cells in AA lesions, providing *in situ* evidence for MIF-mediated crosstalk between fibroblasts and dendritic cells (Figures 8A,B).

## Discussion

Alopecia areata (AA), while often perceived as a cosmetic issue, substantially impairs patients’ psychosocial well-being and quality of life (10, 11). Despite extensive research on adaptive immunity in AA, the roles of other skin-resident cells remain poorly understood. In particular, fibroblasts—along with epithelial-derived cells and innate immune populations (e.g., dendritic cells)—may contribute to initiating or sustaining the inflammatory milieu. In other inflammatory dermatoses, these cell populations are recognized as active contributors: in psoriasis, keratinocytes drive inflammation through the IL-23/IL-17 axis (12); in systemic sclerosis, fibroblasts are key effector cells in fibrosis (13); and in atopic dermatitis, mast cells and ILC2s drive type 2 inflammation (14). To elucidate the contributions of these understudied cellular players in AA, we performed comparative single-cell analysis of lesional and non-lesional scalp.

Regarding the contribution of immune cells to the AA immune microenvironment, this study revealed a predominantly lymphocytic infiltration in lesions with a significantly higher proportion of CD8 + T1 cells than in normal scalp, reinforcing the established role of CD8 + T cells as key effector cells in the disease (1). In the global clustering analysis, mast cell proportions did not differ significantly between AA and NS, with a slight increase observed in AA. However, upon higher resolution subclustering of immune cells, mast cells were significantly decreased in AA compared with NS. This discrepancy likely arises from the substantial infiltration of other immune cell populations in AA lesions, which increases the denominator—total immune cells—and consequently dilutes the relative proportion of mast cells. Furthermore, activated mast cells in AA may undergo transcriptional shifts that result in their redistribution away from the canonical mast cell cluster during high-resolution clustering. Beyond these analytical considerations, the observed reduction may also reflect disease-stage-specific dynamics characterized by mast cell infiltration followed by degranulation and subsequent loss of detectable cells (15).

Epithelial cells in AA exhibit a dual transcriptional phenotype: impaired hair follicle stem cell (HFSC) function alongside an activated inflammatory signature. HFSCs showed downregulation of stemness markers LGR5 and LHX2, aligning with models of autoimmune disruption of the HFSC niche (16, 17) while downregulated protein folding pathways suggest increased vulnerability to inflammatory cytokines. Concurrently, epithelial cells displayed enhanced MHC class II expression (HLA-DRA, HLA-DRB1), indicating IFN-*γ* exposure and a phenotype consistent with potential antigen-presenting capacity. Elevated HLA-E, MIF, PLCG2, and NUPR1 further reinforce immune privilege collapse (1, 18). Pathway enrichment related to myeloid cell homeostasis and cytokine regulation underscores their active role in shaping the inflammatory microenvironment. Together, these changes illustrate a transition of epithelial cells from immune-privileged to inflammatory participants, though whether this is primary or secondary to T cell attack remains unclear (19).

A key finding of this study is the identification of a novel MIF^+^ fibroblast subset, FB3, enriched in AA lesions. This subset exhibits strong NF-κB activation, responses to oxidative stress and hypoxia, and expression of inflammatory mediators (CD59, B2M, NFKBIA, TNFAIP2, TNFAIP3, NAMPT)—a signature resembling pathogenic fibroblasts in rheumatoid arthritis and inflammatory bowel disease (20, 21). Notably, despite stromal expansion in AA, FB3 shows paradoxical downregulation of fibrotic genes, suggesting functional repolarization away from matrix production toward sustained inflammatory signaling (22). This parallels findings in psoriasis, where SFRP2^+^ inflammatory fibroblasts coordinate immune cell recruitment (23), and in rheumatoid arthritis, where MIF-high fibroblasts with mitochondrial dysfunction are linked to disease persistence (24). The persistence of FB3 in established AA lesions raises the possibility that this subset contributes to disease chronicity. Recent studies have proposed that tissue-resident cells can acquire inflammatory memory (“inflammatory tissue priming”) (25); whether FB3 exhibits such properties in AA warrants further investigation.

Cell-communication analysis revealed that FB3 engages in extensive signaling interactions with innate immune populations, predominantly via the MIF pathway. However, as ligand-receptor inference from transcriptomic data cannot establish causality or directionality, the functional interpretation of these interactions remains correlative. This signaling involves receptor complexes containing CD74, with potential contributions from CD44 and CXCR4 on target cells. Conversely, it is also plausible that inflammatory factors derived from dendritic cells (e.g., IL-1β, TNF-*α*) induce or reinforce the polarization of fibroblasts toward the FB3 phenotype, especially given that FB3 exhibits strong NF-κB and stress response signatures (Figure 5E), which are characteristic of a response to an inflammatory milieu. These findings position FB3 as a key orchestrator of stromal–immune crosstalk, though whether FB3 acts as an initiator or a responder in this crosstalk remains to be determined. This aligns with emerging paradigms in chronic inflammatory skin diseases, where inflammatory fibroblasts serve as central coordinators of immune responses (23). MIF signaling preferentially targets dendritic cells, which in turn express high levels of IL-15—a cytokine critical for maintaining pathogenic CD8^+^ tissue-resident memory T cells (26). However, this proposed FB3–DC–IL-15–CD8^+^ T cell axis is inferred from the literature (26) and remains a hypothesis requiring future experimental validation, as explicitly stated in the Discussion. In summary, this study provides a comprehensive cellular landscape of AA, demonstrating that the inflammatory niche extends beyond immune cells to encompass both epithelial and fibroblast populations. A key finding is the identification of a novel MIF^+^ fibroblast subset, FB3, which is enriched in AA lesions. Based on intercellular communication analyses, we propose a working model in which FB3-derived MIF signals through CD74-containing receptor complexes on dendritic cells, which in turn express IL-15—a cytokine critical for sustaining pathogenic CD8^+^ T cells. However, given the inherent limitations of transcriptome-based interaction inference, the proposed FB3 → dendritic cell → IL-15 axis should be viewed as a hypothesis-generating model. It remains formally possible that dendritic cell-derived signals (e.g., IL-1β, TNF-*α*) contribute to the acquisition or maintenance of the FB3 phenotype, and whether FB3 fibroblasts act as initiators or amplifiers in the disease cascade will require direct functional validation (e.g., cell-type-specific depletion or conditional activation) in future studies. While epithelial cells also exhibit inflammatory reprogramming and immune privilege collapse, whether these changes occur as primary drivers or secondary responses to T cell attack remains to be determined. Together, these findings expand the conceptual framework of AA beyond a T cell-centric disease and position FB3 and the MIF signaling axis as potential targets for therapeutic intervention.

Several limitations should also be acknowledged. The present analysis is based on two public scRNA-seq datasets comprising only 15 samples, which limits statistical power and generalizability. Moreover, the lack of detailed clinical metadata in these public data precludes stratification by AA subtype (e.g., patchy AA, alopecia totalis, alopecia universalis), leaving the heterogeneity of FB3 distribution across disease phenotypes unexplored. The immunofluorescence evidence, while supporting co-localization of MIF and VIM, remains correlative; causality and the cellular origin of the double-positive cells await further experimental validation using MIF inhibition or FB3 depletion in appropriate models. Future studies with larger, well-phenotyped cohorts and functional assays are needed to confirm and extend our observations.

## Data Availability

The datasets presented in this study can be found in online repositories. The names of the repository/repositories and accession number(s) can be found at: https://www.ncbi.nlm.nih.gov/, GSE212450; https://www.ncbi.nlm.nih.gov/, GSE233906.

## Supplementary material

SUPPLEMENTARY FIGURE S1 Quality control and cell type identification in scRNA-seq data. **(A)** Violin plots showing total feature counts, read counts, and the percentages of mitochondrial and ribosomal genes in NS, NL, and AA after quality control. **(B)** Violin plots showing total feature counts, read counts, and the percentages of mitochondrial and ribosomal genes per cluster in scalp scRNA-seq data after quality control. **(C)** UMAP visualization showing the distribution and expression of marker genes across different cell types.

SUPPLEMENTARY FIGURE S2 IL-15 expression predominantly localized to DC3 cells. Among all cell types analyzed—including epithelial-derived cells, immune cells, and stromal cells—IL-15 was found to be mainly secreted by DC3 cells (LAMP3⁺, FSCN1⁺) in the human scalp.

SUPPLEMENTARY FIGURE S3 Expression of stromal markers PI16 and SCARA5 across five fibroblast subtypes. FB3 exhibits distinct stromal cell characteristics, as evidenced by the expression patterns of PI16 and SCARA5 among the five fibroblast subpopulations. (A) the relative abundance of PI16 among the five subpopulations (B) the relative abundance of SCARA5 among the five subpopulations.
