# Supplementary figures and images for: Single-cell landscape of immune remodeling in alopecia areata suggests MIF + fibroblasts and their potential ligand-receptor crosstalk with dendritic cells
Source: Front Med (Lausanne). 2026 May 22;13:1849368. doi: 10.3389/fmed.2026.1849368 (PMC13237675; doi:10.3389/fmed.2026.1849368)

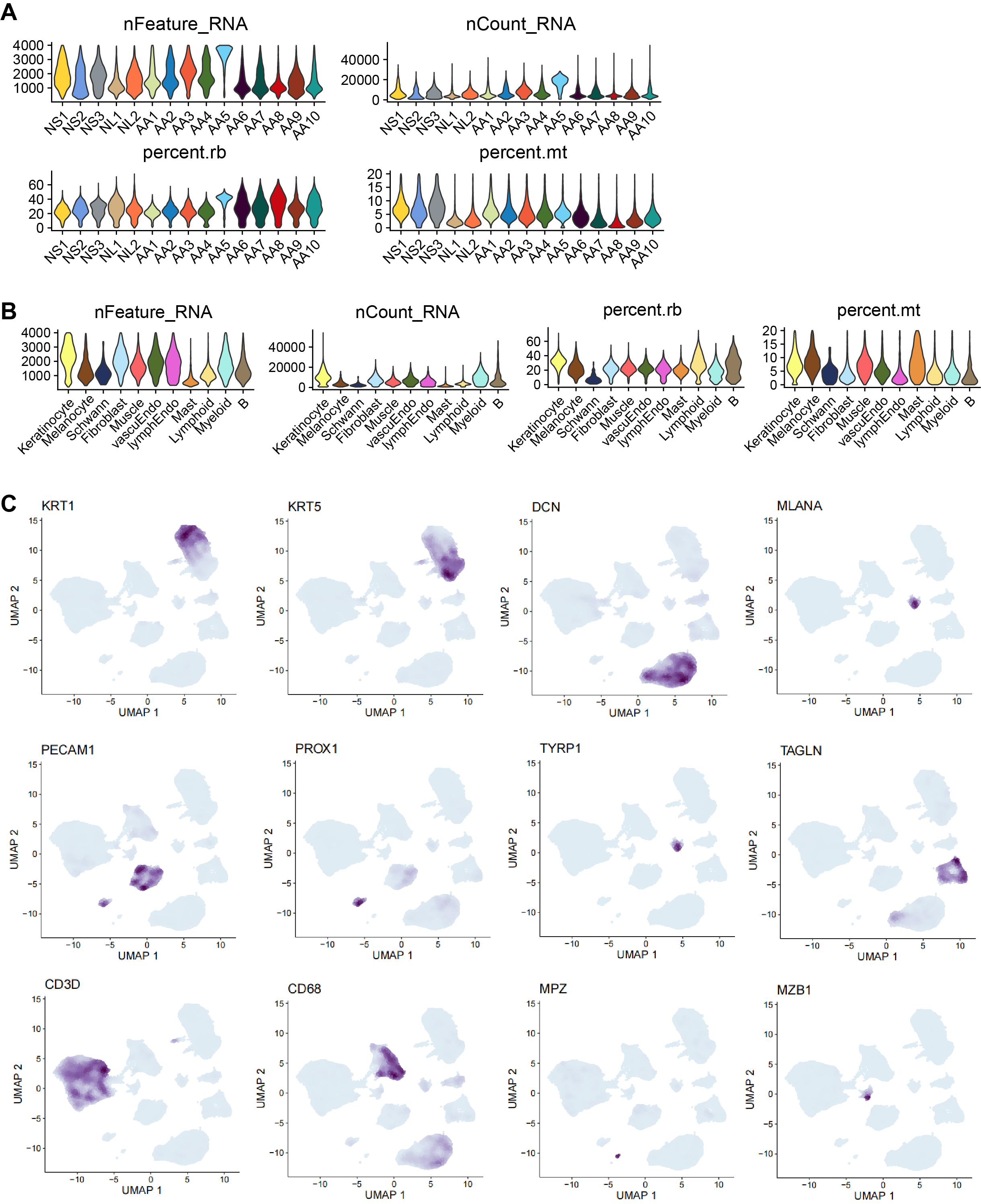

Supplement: SUPPLEMENTARY FIGURE S1 — Quality control and cell type identification in scRNA-seq data. (A) Violin plots showing total feature counts, read counts, and the percentages of mitochondrial and ribosomal genes in NS, NL, and AA after quality control. (B) Violin plots showing total feature counts, read counts, and the percentages of mitochondrial and ribosomal genes per cluster in scalp scRNA-seq data after quality control. (C) UMAP visualization showing the distribution and expression of marker genes across different cell types. [file Image_1.tif]

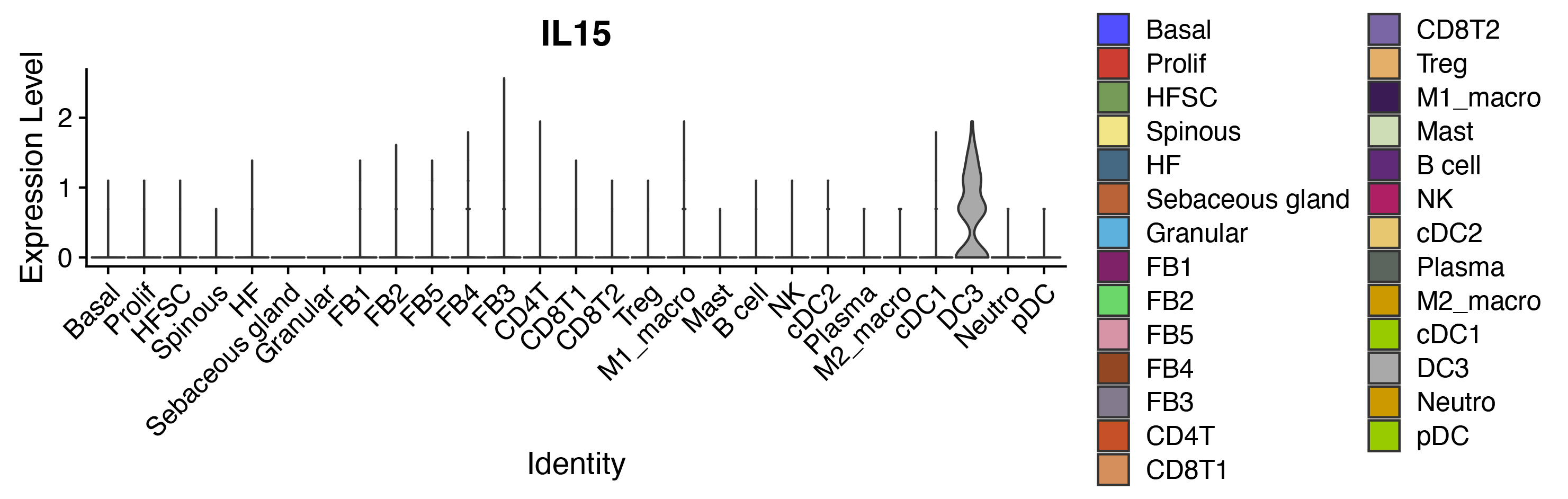

Supplement: SUPPLEMENTARY FIGURE S2 — IL-15 expression predominantly localized to DC3 cells. Among all cell types analyzed—including epithelial-derived cells, immune cells, and stromal cells—IL-15 was found to be mainly secreted by DC3 cells (LAMP3⁺, FSCN1⁺) in the human scalp. [file Image_2.tif]

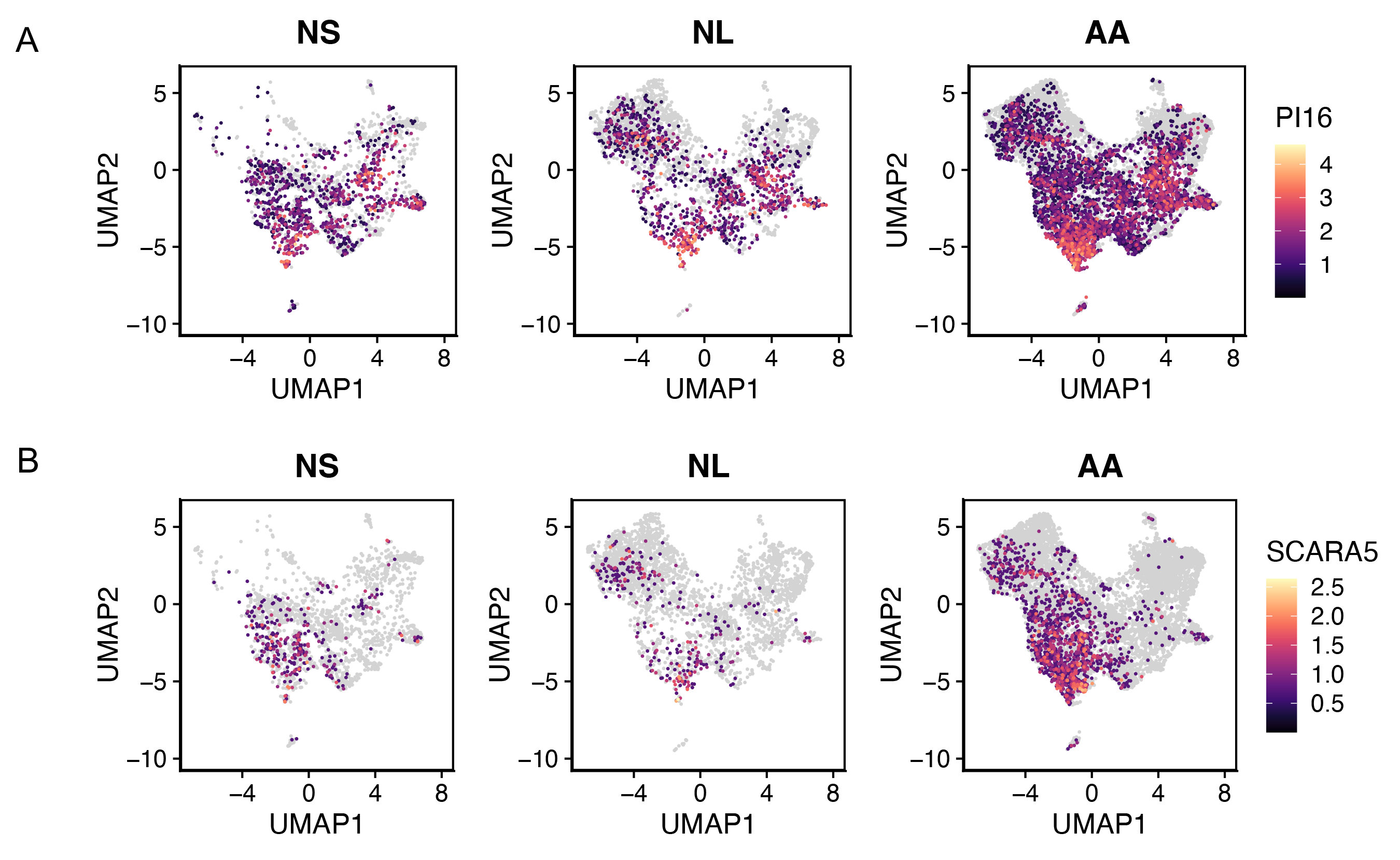

Supplement: SUPPLEMENTARY FIGURE S3 — Expression of stromal markers PI16 and SCARA5 across five fibroblast subtypes. FB3 exhibits distinct stromal cell characteristics, as evidenced by the expression patterns of PI16 and SCARA5 among the five fibroblast subpopulations. (A) the relative abundance of PI16 among the five subpopulations (B) the relative abundance of SCARA5 among the five subpopulations. [file Image_3.tif]
